# Microbial and Chemical Characterization of Natural-Style Green Table Olives from the Gordal, Hojiblanca and Manzanilla Cultivars

**DOI:** 10.3390/foods12122386

**Published:** 2023-06-15

**Authors:** José Luis Ruiz-Barba, Antonio Higinio Sánchez, Antonio López-López, Amparo Cortés-Delgado, Alfredo Montaño

**Affiliations:** Food Biotechnology Department, Instituto de la Grasa (CSIC), Carretera de Utrera, Km. 1, 41013 Seville, Spain; jlruiz@ig.csic.es (J.L.R.-B.); ahiginio@ig.csic.es (A.H.S.); all@cica.es (A.L.-L.); acortes@cica.es (A.C.-D.)

**Keywords:** green table olives, Manzanilla cultivar, Gordal cultivar, Hojiblanca cultivar, microbiota, spontaneous fermentation, metagenomics

## Abstract

Microbial and biochemical changes in the brine during the spontaneous fermentation of Gordal, Hojiblanca and Manzanilla olive cultivars processed according to the natural style were monitored. The microbial composition was assessed through a metagenomic study. Sugars, ethanol, glycerol, organic acids and phenolic compounds were quantified by standard methods. In addition, the volatile profiles, contents of phenolic compounds in the olives and quality parameters of the final products were compared. Fermentation in Gordal brines was conducted by lactic acid bacteria (mainly *Lactobacillus* and *Pediococcus*) and yeasts (mainly *Candida boidinii*, *Candida tropicalis* and *Wickerhamomyces anomalus*). In Hojiblanca and Manzanilla brines, halophilic Gram-negative bacteria (e.g., *Halomonas*, *Allidiomarina* and *Marinobacter*) along with yeasts (mainly, *Saccharomyces*) were responsible for the fermentation. Higher acidity and lower pH values were reached in Gordal brines compared to Hojiblanca and Manzanilla. After 30 days of fermentation, no sugars were detected in Gordal brine, but residual amounts were found in the brines from Hojiblanca (<0.2 g/L glucose) and Manzanilla (2.9 g/L glucose and 0.2 g/L fructose). Lactic acid was the main acid product in Gordal fermentation, whereas citric acid was the predominant organic acid in the Hojiblanca and Manzanilla brines. Manzanilla brine samples showed a greater concentration of phenolic compounds than Hojiblanca and Gordal brines. After a 6-month fermentation, Gordal olives were superior compared to the Hojiblanca and Manzanilla varieties regarding product safety (lower final pH and absence of *Enterobacteriaceae*), content of volatile compounds (richer aroma), content of bitter phenolics (lower content of oleuropein, which resulted in less perceived bitterness) and color parameters (more yellow and lighter color, indicating a higher visual appraisal). The results of the present study will contribute to a better understanding of each fermentation process and could help to promote natural-style elaborations using the above-mentioned olive cultivars.

## 1. Introduction

Table olives are one of the most popular fermented foods in the Mediterranean area, mainly Spain, Italy and Greece. According to the Association of Exporters and Industrialists of Table Olives (ASEMESA), the average production of table olives in Spain in the last five seasons was 561,100 tons, which represents 19.7% of the world production of this product [1]. The most important olive cultivars dedicated to green table olive production in Spain are Hojiblanca (46% of the total Spanish production), Manzanilla (36%) and Gordal (7%) [2]. The Hojiblanca (*Olea europaea arolensis*) olive is located in the provinces of Cordoba and Malaga and, to a lesser extent, in those of Seville and Granada. It obtained its name from the whitish color on the underside of its leaves and has double aptitude, presenting good characteristics for the production of olive oil and table olives. The fruit has a regular shape and its size varies greatly, ranging from 230 to 700 olives/kg. The Manzanilla (*Olea europaea pomiformis*) olive, also known as Manzanillo, is cultivated widely throughout Spain, mainly in the province of Seville. This variety of olive is characterized by its high productivity and harvest, which is conducted by hand, to avoid any damage to the fruit. The Gordal (*Olea europaea regalis*) olive, internationally known as Sevillano, is mainly cultivated in Andalusia, particularly in the province of Seville. It is a vigorous cultivar appreciated mainly for the size of its fruits that reach an average weight of 12.5 g [3]. The main characteristics of these varieties are: Hojiblanca: size, highly variable (230–700 olives/kg); flesh-to-stone ratio, 5:1 to 6.5:1; shape, regular; oil content, 23–29% of fresh weight (f.w.); Manzanilla: size, medium (200–280 olives/kg); flesh-to-stone ratio, 6:1; shape, apple-like; oil content, around 15% f.w.; and Gordal: size, large (100–120 olives/kg); flesh-to-stone ratio, 7.5:1; shape, ellipsoidal: oil content, <10% f.w. [4].

The above-mentioned olive cultivars, when marketed as green table olives, are mainly processed according to the Spanish-style method, which includes an alkaline treatment and washing with water prior brining. Surprisingly, the industrial production of natural-style olives is relatively scarce, in spite of this type of table olives being very appreciated by consumers and being popular in different areas using autochthonous olive cultivars (e.g., Gordal in the province of Seville, Arbequine in the region of Catalonia and Aloreña in the province of Málaga). In addition, the natural-style table olive is the only one recognized as an organic product, since it only uses olives, water and common salt as raw materials; it is nutritionally superior to the Spanish style due to its higher content of poliphenols, sterols, total fatty acids fractions and fiber [5]. 

In general, the natural fermentation process is conducted by the indigenous microbiota, mainly yeast, without the addition of starters [6]. Physicochemical conditions and olive cultivar strongly affect the growth of lactic acid bacteria (LAB) in the natural-style table olives [7]. Recently, we made a comprehensive study of the microbial and biochemical changes during the spontaneous fermentation of the natural-style green olives from the Manzanilla cultivar [8]. This cultivar is known to present relatively high concentrations of polyphenols (mainly represented by oleuropein, which is responsible for the bitterness taste), which appears to be the main reason for the usual absence of LAB growth throughout the fermentation in this type of elaboration [9]. Furthermore, this fact makes the fermentation time required for an acceptable debittering particularly long (>8 months), since the debittering process relies on diffusion phenomena and on the presence of yeast species with β-glucosidase and esterase activities, but it lacks the contribution of LAB species with such enzymatic activities [10]. In this sense, the Gordal and Hojiblanca cultivars could be more adequate for the natural-style elaboration, as these cultivars present a lower content of oleuropein than Manzanilla [11]. However, to the best of our knowledge, no detailed study on the natural fermentation of these two varieties has been published to date.

In addition to polyphenols, the profile of volatile compounds, generally related with the aroma in foodstuffs, is important to evaluate the quality of the final product. Hence, different research groups included the analysis of volatile compounds in their studies on table olives from different olive cultivars in recent years [9,12,13,14,15,16,17,18,19]. The objectives of the present work are to study the microbial community dynamics and biochemical changes in the brines of the natural-style fermentation using the cultivars Gordal, Hojiblanca and Manzanilla, and to compare the main characteristics of the final products, including the contents of volatile compounds, polyphenols and quality parameters. The results of the present study will contribute to a better understanding of each fermentation process and could help to promote the natural-style elaboration using the above-mentioned olive cultivars. This can have a positive impact both on the consumer (consumption of organic food with a high content of beneficial nutrients) and on the industry (lower production costs and a smaller volume of wastewater than the Spanish-style elaboration). 

## 2. Materials and Methods

### 2.1. Olive Processing

The olives (Gordal, Hojiblanca and Manzanilla cultivars) were harvested in Arahal (Seville province, Spain) at their mature-green stage (Gordal, on 14 September; Manzanilla, on 28 September; and Hojiblanca, on 14 October 2021–2022 season) and transported to our laboratories to be processed. Olives from each cultivar were subjected to quality control to remove damaged fruits and then placed into cylindrical plastic vessels (3.3 kg of fruits plus 2.1 L of liquid each). Then, the olives were directly immersed in a brine containing 10% (*w*/*v*) of NaCl. For each cultivar, fermentations were conducted in duplicate at ambient temperature, which was in the range of 8–22 °C. 

### 2.2. Sampling

Brine samples from each vessel were taken during fermentation to control the main chemical and microbiological characteristics. Microbial DNA extraction from the fermenting brines were conducted at days 60, 120 and 180. Non-volatile compounds, including sugars, organic acids and phenolic compounds, in brine were analyzed at days 30, 60, 120 and 180. In addition, at the final sampling, brines were analyzed for volatile compounds and olive samples were analyzed for phenolic compounds, color parameters and firmness.

### 2.3. Chemical and Microbiological Analyses in Brine

#### 2.3.1. Physicochemical Analyses

The titratable acidity, pH, combined acidity and salt content of the olive brines were determined as described in [20]. A Metrohm 670 titroprocessor (Herisau, Switzerland) was used for the measurements of pH, titratable acidity and combined acidity. The titratable acidity was determined by titrating up to pH 8.3 with 0.2 N NaOH and expressed as g/100 mL of lactic acid. The combined acidity was determined with 2 N HCl until the pH value reached 2.6 and was expressed as the equivalent of NaOH per liter. Sodium chloride was determined by titration with silver nitrate.

#### 2.3.2. Analysis of Sugars, Organic Acids, Ethanol and Glycerol

Major sugars in brines (glucose, fructose, mannitol and sucrose) were determined by HPLC following a previous method [21] with modifications. A brine sample (0.25 mL) and 0.75 mL of internal standard (xylitol, 2 g/L) were applied to an SPE cartridge (Sep-Pak Vac, tC18, 500 mg, Waters) that had been previously conditioned with methanol (2 mL) and water (10 mL). The SPE eluate was collected in a test tube, and then 1 mL water was applied to the cartridge and the eluate was collected in the same test tube. The solution was then desalted by adding 1 g of a strongly acidic resin (Amberlite IR-120, Sigma-Aldrich, St. Louis, MO, USA) and 1 g of a weakly basic resin (Amberlite IRA-96, Sigma-Aldrich, St. Louis, MO, USA). Samples were shaken occasionally during a 60 min desalting period. Finally, an aliquot of the solution was filtered through a 0.45 µm nylon filter, and 50 µL of the filtrate was injected into the chromatograph. The separation was performed on a Rezex RCM Monosaccharide column (300 × 7.8 mm i.d., Phenomenex, Torrance, CA, USA) at 80 °C, using deionized water as the mobile phase at a flow rate of 0.6 mL/min and a refractive index detector.

Lactic acid, acetic acid, citric acid, succinic acid, ethanol and glycerol were quantified by HPLC following a previous method [22] with modifications. The separation was conducted on a Rezex ROA-Organic Acid H+ (300 × 7.8 mm i.d.) column (Phenomenex, Torrance, CA, USA) held at 50 °C, using 0.005 M H_2_SO_4_ as the mobile phase at a flow rate of 0.6 mL/min and a refractive index detector. Brine samples (20 µL) were directly injected into the chromatograph after filtration through a 0.45 µm nylon filter. Concentrations were calculated by the comparison of the peak areas with those of the external standards for each compound. All samples were analyzed in duplicate.

#### 2.3.3. Analysis of Phenolic Compounds

Phenolic compounds (oleuropein, hydroxytyrosol, tyrosol and verbascoside) in brine were analyzed by HPLC. A mixture of 0.2 mL of brine, 0.1 mL of internal standard (100 mg/L gallic acid) and 0.2 mL of water was filtered through a 0.45 µm pore size nylon filter and an aliquot (20 µL) was injected into the chromatograph. The HPLC system consisted of a Waters 2695 separation module (Waters Assoc., Milford, MA, USA) connected to a Waters 996 photodiode array detector and controlled with Empower 2 software (Waters). The chromatographic conditions were as follows: column, Luna Phenyl-Hexyl (5 µm, 250 × 4.6 mm, Phenomenex, Torrance, CA, USA); column temperature, 35 °C; flow rate, 1 mL/min; mobile phase, (A) water adjusted to pH 2.0 with phosphoric acid and (B) methanol; gradient solvent program, 0–10 min, 90% A to 70% A; 10–30 min, 70% A; 30–40 min, 70% A to 60% A; 40–45 min, 60% A; 45–50 min, 60% A to 50% A; 50–66 min, 50% A to 10% A; and 66–67 min, 10% A to 90% A. The detection of phenolic compounds was conducted at 280 nm. All phenolic compounds were identified by comparing their retention times and UV-visible spectra to those of authentic standards. For quantification by the external standard method, the calibration curves of each phenolic compound in methanol were used.

#### 2.3.4. Analysis of Volatile Compounds

The volatile compounds were determined by headspace solid-phase microextraction combined with gas chromatography-mass spectrometry (HS-SPME/GC-MS). Briefly, 2 mL of brine was placed into a 15 mL glass vial with 20 µL of internal standard (6-chloro-2-hexanone, 20 mg/L) and volatiles were extracted, identified and quantified according to the method described in [9] with several modifications (1 cm–80 µm Divinylbenzene/Carbon Wide Range/Polydimethylsiloxane (DVB/CWR/PDMS) fiber (Agilent Technologies, Santa Clara, CA, USA), SPME autosampler (PAL3, Agilent), desorption time of 5 min). The volatile compounds were semi-quantified by the comparison of the peak areas to those of the internal standard (6-chloro-2-hexanone). Each sample was analyzed in duplicate.

#### 2.3.5. Microbial Counts during Fermentations

The populations of the main groups of microorganisms were determined by plating the brines and their decimal dilutions (in 0.9% NaCl) on the appropriate solid media with a spiral plater (Easy Spiral Dilute, Interscience, Saint Nom la Bretèche, France). The culture media used were de Man–Rogosa–Sharpe agar (MRS, Oxoid, Basingstoke, UK) supplemented with 0.02% (*w*/*v*) sodium azide (Sigma-Aldrich, St. Louis, MO, USA) and 0.05% (*w*/*v*) L-cysteine (AppliChem GmbH, Darmstadt, Germany) for LAB; glucose-yeast extract agar containing oxytetracycline (0.01% *w*/*v*; AppliChem; OGYE) for yeast; VRBG agar (Laboratorios Conda S.A., Torrejón de Ardoz, Spain) for *Enterobacteriaceae*; and plate count agar (PCA, Labkem, Premià de Dalt, Spain) for mesophilic aerobic bacteria (MAB). MRS plates were incubated under anaerobic conditions using a DG250 Anaerobic Workstation (Don Whitley Scientific Ltd., Shipley, West Yorkshire, UK), with a gas mixture consisting of 10% H_2_-10% CO_2_-80% N_2_ at 30 °C for 72 h. OGYE plates were incubated aerobically at 25 °C for 72 h. VRBG plates were incubated aerobically at 37 °C for 24 h. PCA plates were incubated aerobically at 22 °C for 72 h. The resulting numbers of colony-forming units were counted with a Scan 500 (Interscience, Saint-Nom-la-Bretèche, France) colony counter. The detection limit was established in 10 CFU/mL.

#### 2.3.6. Metagenomic Analysis

At each sampling point, 200 mL of the fermenting olive brines were collected and centrifuged to obtain a microbial pellet. The pellets were washed with 6% (*w*/*v*) NaCl and centrifuged again to obtain a final pellet, which was stored at −30 °C until use. Total DNA from the preserved, defrosted pellets was extracted and purified using the DNeasy PowerFood Microbial Kit (Qiagen, Germantown, MD, USA). The metagenomic analyses were conducted as described in a previous work [8]. The distribution of reads in the quality check protocol and DADA2 routine is shown in Table S1.

### 2.4. Analyses in Olive Fruit

#### 2.4.1. Analyses of Phenolic Compounds

The polyphenols in pulp were extracted by liquid–liquid extraction following the procedure of McDonald et al. [23] with some modifications. A homogenized olive pulp (10 g) was placed into a beaker and 50 mL of methanol–water (60:40, *v*/*v*) was added. After 2 min of intense homogenization with Ultra-Turrax (IKA Labortechnik, Staufen, Germany), the paste was filtered and washed several times with methanol–water (60:40, *v*/*v*) until 100 mL of the extract was collected. An aliquot of the extract (20 mL) was extracted with hexane (2 × 20 mL) to remove oil, and the hydroalcoholic extract was then filtered through a nylon filter (0.45 µm). An aliquot (0.2 mL) of the filtrate was used for analysis by HPLC, for the analysis in brine mentioned above. In addition, this filtrate was analyzed for total phenols using a Folin–Ciocalteu reagent as reported in [24], and the results are expressed as gallic acid equivalents (mg/kg pulp).

#### 2.4.2. Quality Parameters

The color of the olives was measured using a Color-View model 9000 spectrophotometer (BYK-Gardner, Inc., Silver Spring, MD, USA) with a measurement area of 11 mm diameter, 45° circumferential illumination and an observation angle of 0°. All measurements were conducted on the CIE 1976 L*a*b* scale using illuminating conditions CIE type C, 10° observer. For each sample, the results corresponded to the mean of 20 measurements, each made on one olive. Chroma was calculated as [(a*)^2^ + (b*)^2^]^1/2^. Hue angle was calculated as tan^−1^ (b*/a*). The color index *i* was obtained by the following equation: *i* = (4*R*635 + *R*590 − 2*R*560)/3, where *R*635, *R*590 and *R*560 are the values of reflectance at 635, 590 and 560 nm, respectively. 

The firmness of olives was determined using a Kramer shear compression cell coupled with TA.TXplus Texture Analyser (Stable Micro System, Surrey, UK). The cross-head speed was 200 mm/min. The firmness was the mean of 10 replicate measurements, each of which was performed on three pitted olives, and expressed as Newton/100 g pitted olives. 

The sensory analysis of the olive bitterness was evaluated by 14 members of the sensorial panel of the Instituto de la Grasa staff. The perceived bitter taste was quantified on a non-structured line scale from 1 (absence of bitterness) to 11 (strong presence of the bitter taste). The following caffeine solutions were used as reference: 40 mg/mL and 100 mg/mL, corresponding to 4 5 and 7 5 scores on the unstructured scale. Each sample (3–5 olives) was served to panelists in a cupping glass, which were coded with three randomly chosen digits, in separate booths in conditions of normal daylight and room temperature. Between the tests, the panelists were provided with tap water to cleanse the palate. Samples were analyzed in triplicate. The results corresponded to mean scores (panel average).

### 2.5. Statistical Analyses

A one-way analysis of variance (ANOVA, Duncan’s test) was applied to the collected data for microbial counts, volatile compounds, phenolic compounds, color parameters and firmness in order to determine differences among cultivars or sampling times. Significant differences were determined at the *p* < 0.05 level. The ANOVA was performed using SPSS software v. 23.0 (IBM Corp., Armonk, NY, USA). 

## 3. Results and Discussion

### 3.1. Changes in Microbiological Counts and Physicochemical Characteristics during Fermentation

The microbiological counts are shown in Table 1. *Enterobacteriaceae* were not detected in any sample, while LAB growth was only detected in the fermenting brines of the Gordal cultivar. In contrast, yeasts were present in all samples, being the dominant microbial group in the brines of the Hojiblanca and Manzanilla cultivars. In contrast, LAB were dominant in the Gordal brines, so that after 60 days, their populations reached 6.2 ± 0.4 log CFU/mL and hardly changed for the rest of the fermentation. The absence of LAB growth in the Manzanilla brines was somewhat expected, as it is supported by previous studies that point to the higher content in antimicrobial compounds derived from phenolics as the main cause [9,25,26,27]. In the case of the Hojiblanca variety, seasonal variations in the content of essential nutrients and/or the presence of high concentrations of natural inhibitory compounds (mainly phenolics) in the fruits used for the experiment could contribute to the absence of LAB growth in this cultivar. It has been reported that the phenolic content of the olive fruits increases from the Gordal variety (low) to Hojiblanca (medium) and Manzanilla (high) [11]. The yeast population showed similar counts in the three fermentations after 60 days (around 5.7 log CFU/mL) with a slight decrease (1 log in Gordal and lower than 1 log in Manzanilla and Hojiblanca) afterwards. The counts in PCA for MAB were virtually the same as those of the dominant group for each olive variety: LAB in Gordal or yeast in the Hojiblanca and Manzanilla brines, indicating that these dominant microorganisms were the main responsible microorganisms for the actual counts observed in this non-selective culture medium, which allows for the growth of both bacteria and yeast on it.

The changes in the physicochemical characteristics of brines are shown in Table 2. Higher acidity and lower pH values were reached in the Gordal brines compared to Hojiblanca and Manzanilla (0.91% acidity–pH 3.46 in Gordal versus 0.34–4.66% and 0.42–4.61% in Hojiblanca and Manzanilla, respectively, after 180 days of fermentation). Gordal brines showed these acidity and pH values due to the substantial growth of LAB from a very early stage in the fermentation process, in contrast to their absence in the brines of the two other varieties. The combined acidity, which refers to the organic acid salts present in brine, was significantly lower (e.g., 2-fold lower at day 30) in Gordal than in Hojiblanca or Manzanilla. As a consequence, the Gordal brine presented a lower buffer capacity, which explains that, with the same titratable acidity, the pH obtained is lower in this olive variety (see data at day 60). The concentration of NaCl in the brines reached final values of 5.6–6.0% NaCl in the three olive cultivars. As the required minimum salt concentration for this product is 6.0% [28], the addition of salt might be required in some cases to guarantee product safety. 

It can be also noted that the final pH values in Hojiblanca and Manzanilla olives were slightly over the pH required by the International Olive Council (IOC) normative [28] (i.e., pH < 4.3), although the final titratable acidity values were above the required value (0.3% as lactic acid). Therefore, in order to improve the product safety by reaching pH values lower than 4.3, the addition of lactic acid into the initial brine, as previously conducted by Aponte et al. [25], or into the fermenting brine at the beginning of the fermentation would be advisable in natural-style green olives from the Hojiblanca and Manzanilla cultivars. However, such acidification could affect the yeast biota [29]. Further research is needed to confirm this point.

### 3.2. Changes in Fermentation Substrates and Major End-Products

**Figure 1:**
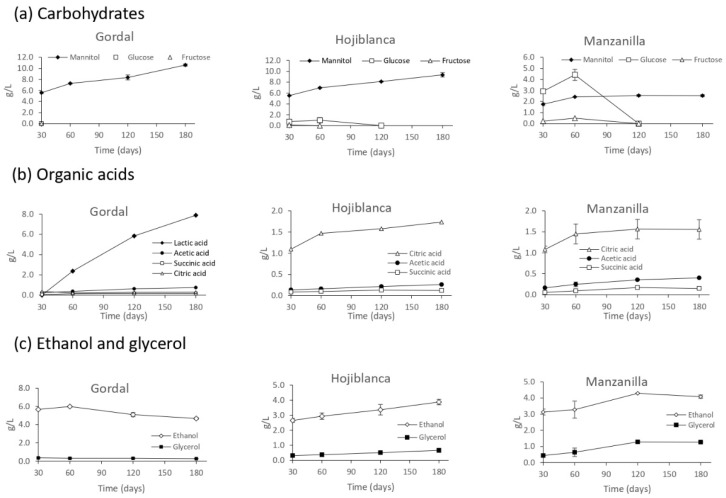
Changes in the composition of (**a**) carbohydrates, (**b**) organic acids and (**c**) ethanol and glycerol during the fermentations of Gordal, Hojiblanca and Manzanilla olive cultivars processed in the natural style. Points are means of duplicate fermentations. Where error bars are not visible, determinations were within the range of the symbols on the graph.

The concentrations of single carbohydrates in raw olives (g/100 g wet pulp) were as follows: 0.2% sucrose, 4.5% glucose, 0.9% fructose and 2.2% mannitol in Gordal; 0.3% sucrose, 2.2% glucose, 0.2% fructose and 1.3% mannitol in Hojiblanca; and 0.4% sucrose, 4.0% glucose, 0.9% fructose and 0.7% mannitol in Manzanilla. These carbohydrates diffused into the brines and were subsequently metabolized during fermentations through the microbial activity. Sucrose was not detected in brine in any case. While mannitol was not consumed in any case, which is in accordance with our own previous studies [8,9], glucose and fructose were metabolized differently by the microbiota growing in the brines of each olive variety. As shown in Figure 1a, the highest consumption rate of these sugars was observed in the Gordal variety, so that no glucose or fructose was detected after 30 days of fermentation. At this sampling time, small amounts of glucose (<0.2 g/L) were measured in the Hojiblanca brine, whereas 2.9 g/L glucose and 0.2 g/L fructose were still found in the Manzanilla brine. The presence of LAB at high numbers early in the fermentation of the Gordal variety is most probably the cause for such a quick sugar consumption. Instead, the low levels of sugar observed for the Hojiblanca variety after 30 days of fermentation could be explained by the low initial content of sugars in the fruits in addition to yeast metabolism. 

**Figure 2:**
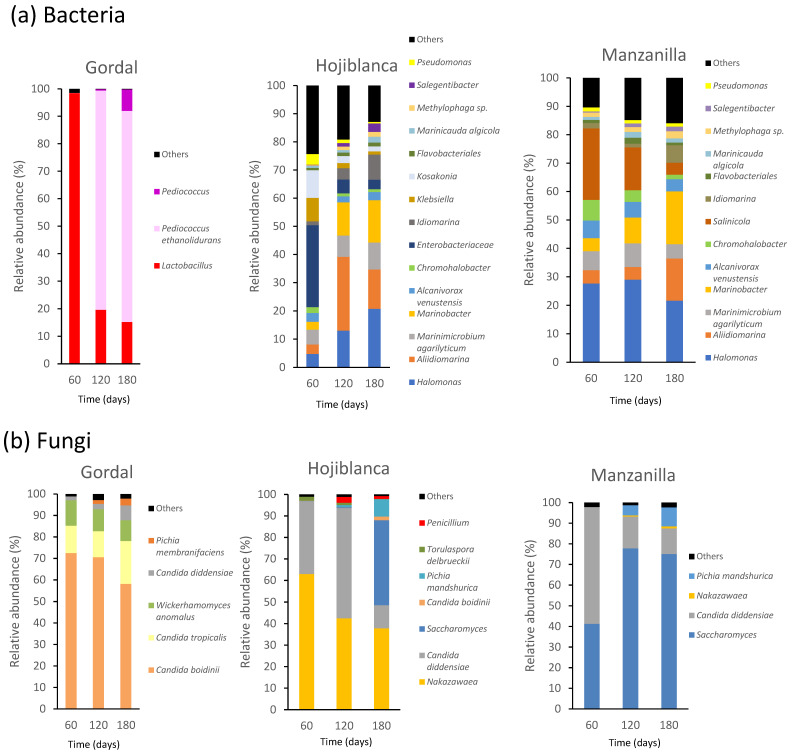
Evolution of (**a**) bacteria and (**b**) fungi in brines from the Gordal, Hojiblanca and Manzanilla cultivars processed in the natural style. Only microbial OTUs with abundances >1% in at least 2 samples were considered. The abundance of OTUs in the 2 biological replicates was averaged.

The major organic acids produced in the different fermentations are shown in Figure 1b. Lactic acid was only detected in the Gordal fermentations, being the major end-product, followed by acetic and citric acids, reaching final concentrations of 7.9, 0.8 and 0.3 g/L, respectively. This result clearly reflects the lactic acid fermentation conducted by the LAB of the genera *Lactobacillus* and *Pediococcus* detected through the metagenomic study (Figure 2). In contrast, citric acid was the predominant organic acid produced in the Hojiblanca and Manzanilla brines, reaching final concentrations of 1.8 and 1.6 g/L, respectively, whereas lactic acid was detected in trace amounts (<0.02 g/L). In the absence of LAB, explaining the lack of lactic acid production, yeast genera, such as *Candida*, *Pichia* and *Saccharomyces*, detected in high proportions in these brines (Figure 2), are known to produce citric acid [30]. As no acetic acid bacteria were identified in the metagenomic study, acetic acid must be the physiological by-product of the alcoholic fermentation conducted by yeast, such as *Saccharomyces* [31], which was present in these brines (Figure 2). Finally, succinic acid was found at low concentrations (<0.2 g/L) in all the three olive fermentations.

Other major end-products, such as ethanol, reached final concentrations of 4.7, 3.9 and 4.1 g/L in the Gordal, Hojiblanca and Manzanilla brines, respectively (Figure 1c). Glycerol was also detected in these brines at the final concentrations of 0.3, 0.7 and 1.3 g/L, respectively. The formation of ethanol, glycerol, succinic acid, citric acid and acetic acid in the Hojiblanca and Manzanilla olives can be attributed to sugar metabolism by yeasts [32,33,34]. However, in the Gordal olives, LAB could also contribute to the formation of acetic acid [35].

### 3.3. Changes in Phenolic Compounds in Brines during Fermentation

The concentrations of the main phenolic compounds (hydroxytyrosol, tyrosol, oleuropein and verbascoside) in brines exhibited notable differences between cultivars during fermentation (Table 3). The Manzanilla brine samples showed a greater concentration of phenolic compounds than the Hojiblanca and Gordal brines. In particular, in Manzanilla, the main phenolic compound throughout fermentation was oleuropein, the bitter-tasting secoroid glucoside, reaching a maximum value of 2262 ± 31 mg/L after 120 days and then slightly decreased by the end of the fermentation. However, oleuropein reached about 100 mg/L in Hojiblanca, while it was undetectable in the Gordal brines. These differences in oleuropein concentrations in brine are consistent with the higher bitterness of Manzanilla compared to other Spanish cultivars, such as Hojiblanca, Gordal and Aloreña [36]. Hydroxytyrosol predominated throughout the fermentation process in the Hojiblanca and Gordal cultivars, which is in agreement with previous studies using other olive cultivars, such as Cellina di Nardò, Leccino, Coservolea and Kalamàta [37,38]. The hydroxytyrosol concentration increased gradually with time and reached final concentrations of 471 ± 12, 973 ± 16 and 1004 ± 37 mg/L in the Gordal, Hojiblanca and Manzanilla brines, respectively. The Tyrosol concentration, similarly to hydroxytyrosol, increased gradually during fermentation, reaching 117 ± 1, 103 ± 2 and 139 ± 4 mg/L in the Gordal, Hojiblanca and Manzanilla brines, respectively, at the end of the process. Finally, a similar trend was observed for verbascoside concentration, but with higher values in Hojiblanca than in the Manzanilla and Gordal brines (final concentration of 346 ± 7 mg/L versus 197 ± 10 and 43 ± 2 mg/L, respectively). The above-mentioned increases in concentrations of hydroxytyrosol, tyrosol and verbascoside in brines during fermentations correspond to the progressive decreases in the content of such phenolic compounds in the olives, as demonstrated by other authors [16,39]. The accumulations of hydroxytyrosol and tyrosol in the brines can be attributed to the hydrolysis of oleuropein, hydroxytyrosol-4-β-glucoside and salidroside in the pulp and then diffusion from the pulp into the brine [40]. 

### 3.4. Microbial Communities during Fermentation

The relative abundance (%) of bacteria and fungi in the brines from the three olive varieties processed in the natural-style in relation to fermentation time is shown in Figure 2. Only microbial OTUs with abundances >1% in at least two samples were considered. The number of reads, number of OTUs (genus level) and Good’s coverage of the metataxonomy analyses conducted are shown in Table S2. The data for this study were deposited in the European Nucleotide Archive (ENA) at EMBL-EBI under accession number PRJEB61957 (https://www.ebi.ac.uk/ena/browser/view/PRJEB61957, accessed on 16 June 2023).

Regarding bacteria, significative differences were found between the fermentations using the Gordal variety or the Hojiblanca/Manzanilla ones in terms of diversity, being considerably higher in the last two varieties as denoted by the values of different alpha-diversity estimators (Table S2). In addition, as indicated by the Shannon Equitability Index (EH), the evenness of species (similarity in the abundances of the different OTUs detected) in the respective community was considerably higher for the two last mentioned varieties, in contrast with the low values found for the Gordal variety. This was reflected by the dominance of the genera *Lactobacillus* and *Pediococcus* throughout the fermentation using Gordal olives. *Lactobacillus* was the dominant genus at the initial stage and *Pediococcus* (more specifically, the species *P. ethanolidurans*) at the middle and final stages (Figure 2). In contrast, with Hojiblanca or Manzanilla olives, the number of genera detected was considerably larger, all of them being Gram-negative. Virtually all of the identified bacterial genera have been described in the past as halophiles or halotolerant, according to the HaloDom (v. 1.3) webpage (http://halodom.bio.auth.gr/?view=home, accessed on 15 May 2023), most of them from marine or saline environments. Therefore, a more than probable origin in the marine salt used to elaborate the initial brine can be presumed. Although no Archaea was detected in this study, most of the bacterial genera were already described in a previous study using Manzanilla olives fermented in the natural style [8]. Several authors have also reported the presence of a relatively high abundance of halophilic/halotolerant bacteria, such as *Halomonas*, *Salinicola, Marinobacter*, *Aliidiomarina* and *Pseudomonas*, in brines from the natural-style green olives of different cultivars [12,41,42]. Dominance at the end of the fermentations was less clear than with the Gordal variety, with the most abundant bacterial genera being *Halomonas*, *Marinobacter* and *Alidiomarina* (relative abundances of 21–22%, 15–19% and 14–15%, respectively). Strikingly, *Enterobacteriaceae*, including the genera *Klebsiella*, *Kosakonia* and *Pseudomonas*, counted for 51% of the total OTUs in the Hojiblanca fermentations after 60 days, although its presence was reduced to 6.7% at the end. However, as mentioned above in Section 3.1, no viable *Enterobacteriaceae* were detected in the specific culture medium used in this study (i.e., VRBG). This could be due to an *Enterobacteriaceae* population existing below the detection limit of the technique (10 CFU/mL) or to their lack of viability. In addition, up to 200 mL of brine were used to obtain enough microbial pellets to extract the total DNA for the metagenomic studies, more than two magnitude orders higher than the amount used for the microbial analyses on culture media plates. In contrast, the presence of *Enterobacteriaceae* in the Manzanilla fermentations was very residual (ca. 1.1%). The detection of *Pseudomonas* in both olive cultivars, although at low abundances, is noteworthy, especially when the final pH did not reach a safe value spontaneously. Since this fact could negatively affect the product safety, the addition of lactic acid, as mentioned in Section 3.1, would be an advisable step in the processing of natural-style green olives from the Hojiblanca and Manzanilla cultivars. Further research is needed to design appropriate processing strategies. 

In contrast to what it was found for bacteria, diversity indexes regarding fungi were quite similar for the three olive varieties used, although clear differences were found in the actual species composition (Figure 2). In this regard, *Candida boidinii* was the dominant yeast species throughout the Gordal fermentations, along with *Candida tropicalis* and *Wickerhamomyces anomalus*. Instead, the Hojiblanca and Manzanilla fermentations showed similar yeast genera compositions, both different from the Gordal fermentations, although their evolution and abundance differed considerably. Thus, *Nakazawaea* was relatively abundant in Hojiblanca (38% at the end of fermentation), but was hardly detected in Manzanilla (1%). *Saccharomyces* was abundant in both cultivars, mainly in Manzanilla (75%). It can be also noted that the species *C. diddensiae* was abundant at the beginning in both cultivars (34–57%) and decreased afterwards (11–12% at the end of fermentation). Finally, the EH indexes showed relatively low values, indicating that the abundance of the different species detected was quite uneven during the fermentation, a result of the presence of a few dominant species.

### 3.5. Volatile Compounds

**Figure 3:**
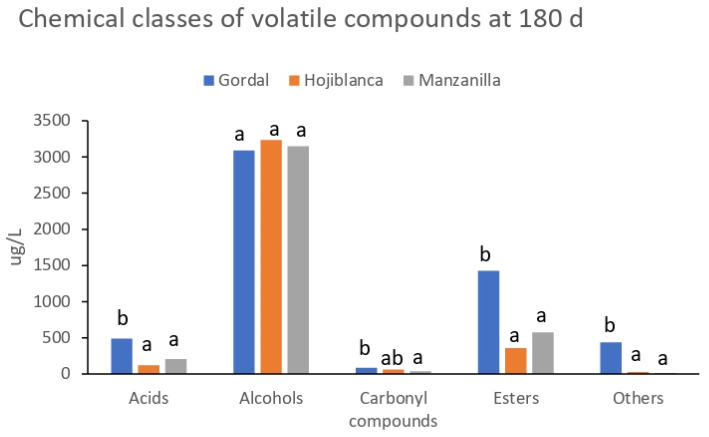
Total contents of the chemical classes of volatile compounds in the fermenting brines of olives from the Gordal, Hojiblanca and Manzanilla cultivars processed in the natural style at the end of fermentation (180 days). Values are means of 2 biological replicates. For each chemical class, different letters above the bars indicate statistically significant difference at *p* < 0.05.

The analysis of volatile compounds at the end of fermentation (180 days) allowed for the identification of 65 different volatiles, of which 45, 40 and 35 were found in brines from the Gordal, Hojiblanca and Manzanilla cultivars, respectively (Figure S1). The volatiles were grouped into different chemical classes, i.e., acids, alcohols, carbonyl compounds, esters, phenols and other compounds, and the composition of the fermenting brine for each olive variety is shown in Figure 3. The total contents of these chemical classes presented higher values in the Gordal brines in comparison with the Hojiblanca and Manzanilla ones, with the exception of alcohols, which did not show significant differences between cultivars. The predominant compound in the Gordal brines was ethanol, followed by ethyl acetate, isopentanol, phenylethyl alcohol, ethyl lactate and acetic acid (Figure S1). It is well-known that ethanol is an important flavor compound formed via the Embden–Meyerhof–Parnas glycolytic pathway. Ethyl acetate and ethyl lactate could be synthesized by the esterification of ethanol with acetyl-CoA and lactyl-CoA, respectively [43,44]. The formation of ethyl lactate is consistent with the high concentrations of lactic acid formed in these olives due to LAB growth. Isopentanol and phenylethyl alcohol could be formed by yeasts from leucine and phenylalanine, respectively, via the Ehrlich pathway [45]. As mentioned in Section 3.2, acetic acid in the Gordal brine could be formed by both yeast and LAB from the sugar metabolism (glycolysis). In the fermenting brines of the Hojiblanca and Manzanilla cultivars, ethanol and isopentanol largely prevailed over all the identified volatiles. This result agrees with our recent study using Manzanilla olives from a previous season processed according to the natural style [8]. ANOVA showed significant differences (*p* < 0.05) between cultivars, with the exception of nonanoic acid, benzaldehyde and isopentyl acetate (Table 4). Focusing on the most abundant volatiles, it can be noted that the concentration of ethanol was of the same magnitude order in the three fermentations, although slightly lower (*p* < 0.05) in the Hojiblanca brines. The isopentanol content was similar in the Manzanilla and Hojiblanca brines, but significantly higher than in Gordal. On the contrary, Gordal exhibited higher contents of ethyl acetate and ethyl lactate. The phenylethyl alcohol content was also higher in Gordal, although not significantly different from that of Hojiblanca. Other relatively abundant compounds were isobutanol in Hojiblanca and methyl 2,5-dimethyl-3-furoate in Manzanilla, although the latter compound presented a high variability between the biological replicates. It is worth mentioning that methyl 2,5-dimethyl-3-furoate was previously found at relatively high levels in Manzanilla olives processed according to the natural style, but it was not detected in those processed according to the Spanish style [8]. Finally, we highlight the relatively high concentrations of creosol and 4-ethylphenol in Gordal, which were not detected neither in Hojiblanca nor in Manzanilla. This could be related to the ability of certain species of *Lactobacillus*, such as *L. plantarum* and *L. pentosus*, to produce volatile phenols from hydroxycinnamic acids (e.g., 4-ethylphenol from p-coumaric acid), although this ability appears to be strain-dependent [14,17]. Among the volatile compounds detected in this study, it is worth mentioning that isopentanol (fermented, alcoholic, banana odor), phenylethyl alcohol (floral, sweet), acetic acid (vinegar) and isobutanol (ethereal, winey) were previously described as aroma-active compounds in table olives [46]. 

### 3.6. Polyphenols and Quality Parameters of Final Products

The analysis of phenolic compounds in the pulp of the fruits at the end of process showed that the content in total phenols (Folin–Ciocalteu) decreased in the following order: Manzanilla > Hojiblanca > Gordal (Table 5). Although correlation studies between phenolic content and bitterness intensity have not been reported in natural-style green table olives, the preliminary results by our group (unpublished data) indicate a significant relationship between the total phenol content (Folin–Ciocalteu method) and the perceived bitterness intensity (sensory panel). Therefore, it is reasonable to infer that the perceived bitterness presents a similar trend as the total phenols. This is in accordance with previous studies on naturally fermented olives from the Manzanilla, Gordal and Hojiblanca cultivars [36]. 

With regard to single phenolic compounds (Table 5), the amount of hydroxytyrosol was significantly lower in Gordal olives (around half) compared to Hojiblanca and Manzanilla, which showed identical contents of hydroxytyrosol (969 mg/kg pulp). The tyrosol content ranged between 132 and 190 mg/kg, with the lowest content in Gordal (*p* < 0.05). The verbascoside content showed a similar trend, but values in Hojiblanca and Manzanilla were 4- to 7-fold higher than those of tyrosol. The oleuropein content was much higher in Manzanilla (7- to 8-fold higher) than in Gordal and Hojiblanca, which did not present statistically significant differences (around 700 mg/kg). It has been reported that the above-mentioned single phenolic compounds have healthy properties, particularly hydroxytyrosol and oleuropein [47]. These results indicate that Gordal olives have the lowest content of phenolic compounds, which affects the organoleptic characteristics (less bitterness) but also the nutritional ones (lower content of bioactive compounds). On the contrary, Hojiblanca and Manzanilla olives are richer in bioactive compounds but also in bitterness. A sensory evaluation of the bitterness intensity on a 10 cm unstructured scale yielded mean scores (panel average) of 3.2, 4.8 and 8.0 for Gordal, Hojiblanca and Manzanilla olives, respectively. This result suggests that, in order to reduce bitterness to acceptable levels in Manzanilla olives (and probably also in Hojiblanca), it would be necessary to apply additional operations (pitting and washing) before packaging. The removal of bitter compounds (mainly represented by oleuropein and its aglycon) could also occur by using starter cultures of yeasts. Ciafardini and Zullo [48] found that the intensity of the bitter taste in natural Taggiasca black table olives was significantly lower for fruits processed with strains of *Candida adriatica*, *Candida diddensiae* and *Wickerhamomyces anomalus* used as starters. Selected *Saccharomyces cerevisiae* strains have been also used as starters in natural Manzanilla black table olives [15], although their impact on olive bitterness was not investigated. Research is under way in our laboratories to evaluate the use of yeast strains as starter cultures for natural Manzanilla and Hojiblanca green olive fermentations.

With regard to other quality parameters, significant differences (*p* < 0.05) in color parameters and firmness were found between the three cultivars at the end of the fermentation (180 days) (Table 5). The values of these characteristics in the present study are similar to those obtained by Ramirez et al. [36]. Gordal olives showed higher values of the parameters L*, b*, chroma and color index, indicating a more yellow and lighter color than Manzanilla and Hojiblanca olives. This means a better visual appraisal in the case of Gordal olives, since it has been found that the higher the color index, the better the sensory evaluation of color in green table olives (at least in Spanish-styles olives) [49]. On the contrary, Gordal olives presented the lowest values in firmness, although differences with Manzanilla were not statistically significant. Even so, as expected, the firmness values were much higher than those in Spanish-style green olives [50]. 

## 4. Conclusions

In this study, microbial community and chemical changes in the brines of natural-style green table olives from the Gordal, Hojiblanca and Manzanilla cultivars were monitored during 6 months of fermentation. The metagenomic analysis showed meaningful differences between the three cultivars, which impacted their physicochemical and biochemical characteristics. Fermentation was conducted by LAB and yeasts in Gordal olives. Instead, halophilic Gram-negative bacteria along with yeasts were the responsible for the fermentation in Hojiblanca and Manzanilla olives. From a safety standpoint, especially as the result of the detection of pathogenic bacteria by the metagenomic analyses, the final physicochemical characteristics of Gordal were more adequate than those of Hojiblanca and Manzanilla. In this sense, in natural-style green olives from Hojiblanca and Manzanilla, we propose the addition of lactic acid to the fermenting brine at the beginning of fermentation in order to compensate for the lack of acidification due to the probable absence of LAB. In addition, Gordal olives were superior to Manzanilla and Hojiblanca with regard to other features related to product quality, such as a richer content of volatile compounds (related to aroma), lower content of bitter phenolics, which resulted in less perceived bitterness, and a better color. Our future research directions will be focused on developing controlled fermentations using starter cultures of bacteria and/or yeasts for the production of high-quality natural-style green table olives, with special emphasis on Hojiblanca and Manzanilla cultivars. The results of the present work will help in the selection of autochthonous strain cultures to achieve this goal.

## Figures and Tables

**Table 1:** Microbial counts (log CFU/mL) in the brines during the fermentation of the Gordal, Hojiblanca and Manzanilla cultivars processed in the natural style *^a^*.

	Gordal	Hojiblanca	Manzanilla
60 days			
Yeast	5.6 ± 0.5 a,B	5.6 ± 0.3 a,B	5.7 ± 0.2 a,B
LAB	6.2 ± 0.4 A	nd	nd
MAB	6.1 ± 0.3 b,A	5.4 ± 0.0 a,B	5.5 ± 0.0 a,A
120 days			
Yeast	4.6 ± 0.1 a,A	5.4 ± 0.1 b,B	5.5 ± 0.2 b,AB
LAB	6.3 ± 0.1 A	nd	nd
MAB	6.4 ± 0.2 b,AB	5.3 ± 0.0 a,B	5.5 ± 0.3 a,A
180 days			
Yeast	4.7 ± 0.1 a,A	5.0 ± 0.0 b,A	5.3 ± 0.1 c,A
LAB	6.7 ± 0.1 B	nd	nd
MAB	6.7 ± 0.1 c,B	4.9 ± 0.1 a,A	5.2 ± 0.1 b,A

*^a^* Values are means ± SD of duplicate fermentations, each analyzed in duplicate (*n* = 4). Means in the same row labelled with different lower-case letters are significantly different (*p* < 0.05). For a given microorganism group, means in the same column labelled with different capital letters are significantly different (*p* < 0.05). nd = not detected (<10 CFU/mL).

**Table 2:** Physico-chemical characteristics of the brines during the fermentation of Gordal, Hojiblanca and Manzanilla cultivars processed in the natural style *^a^*.

	Gordal	Hojiblanca	Manzanilla
30 days			
pH	4.76 ± 0.03 a	4.74 ± 0.05 a	4.75 ± 0.04 a
Titratable acidity (% lactic acid)	0.13 ± 0.00 a	0.21 ± 0.00 b	0.23 ± 0.01 c
Combined acidity (*N*)	0.023 ± 0.000 a	0.047 ± 0.000 b	0.047 ± 0.004 b
Salt content (% NaCl)	6.52 ± 0.09 b	5.74 ± 0.23 a	6.37 ± 0.01 b
60 days			
pH	3.77 ± 0.03 a	4.74 ± 0.01 b	4.70 ± 0.04 b
Titratable acidity (% lactic acid)	0.36 ± 0.01 a	0.36 ± 0.01 a	0.31 ± 0.04 a
Combined acidity (N)	0.030 ± 0.001 a	0.058 ± 0.000 b	0.062 ± 0.006 b
Salt content (% NaCl)	5.83 ± 0.02 a	- *^b^*	6.14 ± 0.08 b
120 days			
pH	3.59 ± 0.02 a	4.68 ± 0.00 b	4.61 ± 0.04 b
Titratable acidity (% lactic acid)	0.72 ± 0.02 c	0.37 ± 0.00 a	0.47 ± 0.01 b
Combined acidity (N)	0.042 ± 0.001 a	0.068 ± 0.000 b	0.071 ± 0.008 b
Salt content (% NaCl)	-	5.66 ± 0.06 a	-
180 days			
pH	3.46 ± 0.00 a	4.66 ± 0.01 b	4.61 ± 0.04 b
Titratable acidity (% lactic acid)	0.91 ± 0.01 c	0.34 ± 0.01 a	0.42 ± 0.02 b
Combined acidity (N)	0.048 ± 0.000 a	0.072 ± 0.000 b	0.073 ± 0.008 b
Salt content (% NaCl)	5.56 ± 0.01 a	5.58 ± 0.06 a	5.98 ± 0.18 b

*^a^* Values are means ± standard deviation of two biological replicates, each analyzed in duplicate. Means in the same row labelled with different letters are significantly different (*p* < 0.05). *^b^* = not analyzed.

**Table 3:** Phenolic compounds in the brines (mg/L) during the fermentation of Gordal, Hojiblanca and Manzanilla natural-style green olives *^a^*.

	Gordal	Hojiblanca	Manzanilla
30 days			
hydroxytyrosol	190 ± 1 a,A	410 ± 7 b,A	428 ± 6 b,A
tyrosol	49 ± 0 a,A	46 ± 4 a,A	69 ± 1 b,A
verbascoside	4 ± 5 a,A	93 ± 12 c,A	67 ± 4 b,A
oleuropein	nd	80 ± 1 a,A	521 ± 60 b,A
60 days			
hydroxytyrosol	295 ± 5 a,B	583 ± 16 b,B	654 ± 1 b,B
tyrosol	68 ± 2 a,B	56 ± 3 a,B	118 ± 9 b,B
verbascoside	17 ± 2 a,B	168 ± 4 c,B	123 ± 4 b,B
oleuropein	nd	105± 12 a,A	1298 ± 107 b,B
120 days			
hydroxytyrosol	403 ± 4 a,C	854 ± 7 b,C	912 ± 10 c,C
tyrosol	94 ± 1 a,C	82 ± 1 a,C	145 ± 7 b,C
verbascoside	30 ± 2 a,C	280 ± 12 c,C	212 ± 30 b,C
oleuropein	nd	105 ± 14 a,A	2262 ± 31 b,C
180 days			
hydroxytyrosol	471 ± 12 a,D	973 ± 16 b,D	1004 ± 37 b,D
tyrosol	107 ± 1 a,D	103 ± 2 a,D	139 ± 4 b,C
verbascoside	43 ± 2 a,D	346 ± 7 c,D	197 ± 10 b,C
oleuropein	nd	93 ± 17 a,A	2044 ± 97 b,C

*^a^* Values are means ± standard deviation of two biological replicates, each analyzed in duplicate. Means in the same row labelled with different lower-case letters are significantly different (*p* < 0.05). For a given compound, means in the same column labelled with different capital letters are significantly different (*p* < 0.05). nd = not detected.

**Table 4:** Concentrations of the volatile compounds in the brine samples from the Gordal, Hojiblanca and Manzanilla cultivars processed in the natural style at the end of fermentation (180 days).

	Gordal		Hojiblanca		Manzanilla	
Volatile compounds ^a^	Mean	SD	Mean	SD	Mean	SD
Acids						
Acetic acid	338 b	24	72 a	7	100 a	15
Isobutanoic acid	9.3	0.8	nd		nd	
Butanoic acid	11.6 b	0.1	nd		4.6 a	0.6
2-Methylbutanoic acid	97 b	19	45 a	5	23.8 a	0.3
Hexanoic acid	13 a	1	nd		50 b	4
Octanoic acid	nd		nd		24	5
Nonanoic acid	12 a	11	3.7 a	0.8	6 a	2
Hydrocinnamic acid	8.6	0.5	nd		nd	
Alcohols						
Ethanol	1572 b	113	1047 a	87	1461 b	59
1-Propanol	nd		5.1 a	0.5	15.9 b	0.4
2-Methyl-3-buten-2-ol	11	1	nd		nd	
Isobutanol	59 a	20	313 b	59	71 a	6
1-Butanol	nd		nd		5.2	0.2
Isopentanol	465 a	70	1067 b	108	1205 b	104
3-Methyl-3-buten-1-ol	8	1	nd		nd	
1-Pentanol	nd		3.5	0.0	nd	
Prenol	17 a	4	6.4 a	0.4	nd	
3-Methyl-1-pentanol	nd		nd		4.9	0.7
1-Hexanol	38 a	9	94 b	2	30 a	1
(Z)-3-Hexen-1-ol	191 b	42	188 b	7	33.5 a	0.9
1-Heptanol	5 a	2	10.1 b	0.7	14.2 c	0.7
2-Ethyl-1-hexanol	6.0 a	0.9	nd		5 a	2
6-Hepten-1-ol	nd		4.3	0.1	nd	
2,3-Butanediol	19	2	nd		nd	
1-Octanol	11 b	2	6.9 a	0.0	7.9 ab	0.4
1-Nonanol	nd		5.1	0.4	nd	
Benzyl alcohol	210 b	32	138 b	32	14 a	3
Phenylethyl alcohol	454 b	29	357 ab	47	279 a	37
3,3,6-Trimethyl-4,5-heptadien-2-ol	11.2	0.1	nd		nd	
Carbonyl compounds						
2-Methylbutanal	nd		7.3 a	0.8	5 a	1
3-Methylbutanal	5.5 a	0.1	11 b	2	4 a	2
3-Pentanone	6.4 a	0.7	4.1 a	0.6	nd	
Acetoin	7.9	1.2	nd		nd	
Nonanal	6 a	1	20 b	5	5.2 a	0.8
Benzaldehyde	8.6 a	0.0	5.0 a	0.6	15 a	6
3-Methylbenzaldehyde	40 b	5	nd		10 a	3
Benzeneacetaldehyde	nd		13.7	0.5	nd	
Esters						
Methyl acetate	22 b	3	11.2 a	0.5	11.0 a	0.4
Ethyl Acetate	634 b	70	216 a	1	303 a	15
Methyl butanoate	nd		3.7	0.3	nd	
Isobutyl acetate	nd		6.2	0.1	nd	
Ethyl butanoate	15	1	nd		nd	
Methyl 2-methylbutanoate	nd		5.5 b	0.4	3.9 a	0.1
Ethyl 2-methylbutanoate	18 b	3	8.1 a	0.1	nd	
Ethyl 3-methylbutanoate	24 b	2	6.6 a	0.6	3.8 a	0.3
Isopentyl acetate	15 a	4	22.1 a	0.7	18 a	1
Ethyl hexanoate	8.6 b	0.3	3 a	2	9 b	1
(Z)-3-Hexenyl acetate	nd		24.3	0.6	nd	
Methyl lactate	11	3	nd		nd	
Ethyl lactate	445	54	nd		nd	
Ethyl octanoate	nd		10 a	5	8 a	1
Ethyl 2-hydroxy-4-methylpentanoate	35	4	nd		nd	
Methyl 2,5-dimethyl-3-furoate	nd		36 a	25	208 a	114
Ethyl 2,4-dimethyl-3-furoate	nd		nd		11	9
Diethyl succinate	19	1	nd		nd	
2-Phenylethyl acetate	7 a	1	8.6 a	0.4	nd	
Methyl hydrocinnamate	9.3	0.1	nd		nd	
Ethyl hydrocinnamate	125	11	nd		nd	
Ethyl (Z)-cinnamate	12	2	nd		nd	
Other compounds						
Dimethyl sulfide	nd		4.8 a	0.6	17 b	2
3-Ethylpyridine	nd		4.8 a	0.8	6.1 a	0.7
o-Guaiacol	nd		14	3	nd	
Creosol	273	13	nd		nd	
Phenol	nd		3.2	0.7	nd	
4-Ethylphenol	159	51	nd		nd	

^a^ Semi-quantitative determination expressed as µg L^−1^ of 6-chloro-2-hexanone. nd = not detected. SD = standard deviation. Means are from two biological replicates, each analyzed in duplicate. Means in the same row labelled with different letters are significantly different (*p* < 0.05).

**Table 5:** Phenolic compounds, color parameters and firmness in Gordal, Hojiblanca and Manzanilla natural-style green olives at the end of fermentation (180 days) *^a^*.

	Gordal	Hojiblanca	Manzanilla
Phenolic compounds (mg/kg)			
Hydroxytyrosol	501 ± 24 a	969 ± 40 b	969 ± 51 b
Tyrosol	132 ± 5 a	190 ± 8 c	162 ± 3 b
Verbascoside	152 ± 14 a	1348 ± 66 c	735 ± 44 b
Oleuropein	637 ± 6 a	729 ± 77 a	4823 ± 355 b
Total phenols *^b^*	4021 ± 187 a	6160 ± 97 b	8427 ± 399 c
Color parameters			
Color index *i*	34.8 ± 0.2 c	24.8 ± 0.5 a	30.9 ± 1.1 b
L*	51.9 ± 0.5 b	45.4 ± 1.0 a	45.3 ± 0.7 a
a*	7.1 ± 0.4 b	5.2 ± 0.4 a	7.7 ± 0.3 b
b*	35.6 ± 0.4 b	26.4 ± 1.0 a	27.9 ± 1.5 a
Chroma	36.3 ± 0.3 b	26.9 ± 0.9 a	28.9 ± 1.5 a
Hue angle	78.5 ± 0.8 b	78.7 ± 1.1 b	74.4 ± 0.4 a
Firmness (N/g)	48.9 ± 0.3 a	57.4 ± 0.3 b	54.4 ± 4.8 ab

*^a^* Values are means ± standard deviation. Values in the same row labelled with different letters are significantly different (*p* < 0.05). *^b^* Total phenols (Folin–Ciocalteu) expressed as gallic acid equivalents.

## Data Availability

The data used to support the findings of this study can be made available by the corresponding author upon request.

## Supplementary Materials

The following supporting information can be downloaded at: https://www.mdpi.com/article/10.3390/foods12122386/s1. Table S1: Distribution of reads in the quality check protocol and DADA2 routine of samples of the fermenting brines of olives subjected to the metataxonomy analysis of 16S rRNA and ITS amplicons; Table S2: Number of reads, OTUs, Good’s coverage and different alpha diversity estimators of the metataxonomy analysis of 16S rRNA (Bacteria and Archaea) and ITS (Fungi) amplicons of the fermenting brines from the Gordal, Hojiblanca and Manzanilla cultivars processed in the natural style; Figure S1: Volatile profiles in the fermenting brines of olives from the Gordal, Hojiblanca and Manzanilla cultivars processed in the natural style at the end of fermentation (180 days). Values are means of 2 biological replicates. Error bars represent standard deviations (*n* = 4).
