# Supplementary material for: Microbial and Chemical Characterization of Natural-Style Green Table Olives from the Gordal, Hojiblanca and Manzanilla Cultivars
Source: Foods. 2023 Jun 15;12(12):2386. doi: 10.3390/foods12122386 (PMC10297402; doi:10.3390/foods12122386)
Supplement: Supplementary file 1 [file foods-12-02386-s001.zip › Figure S1.pdf]

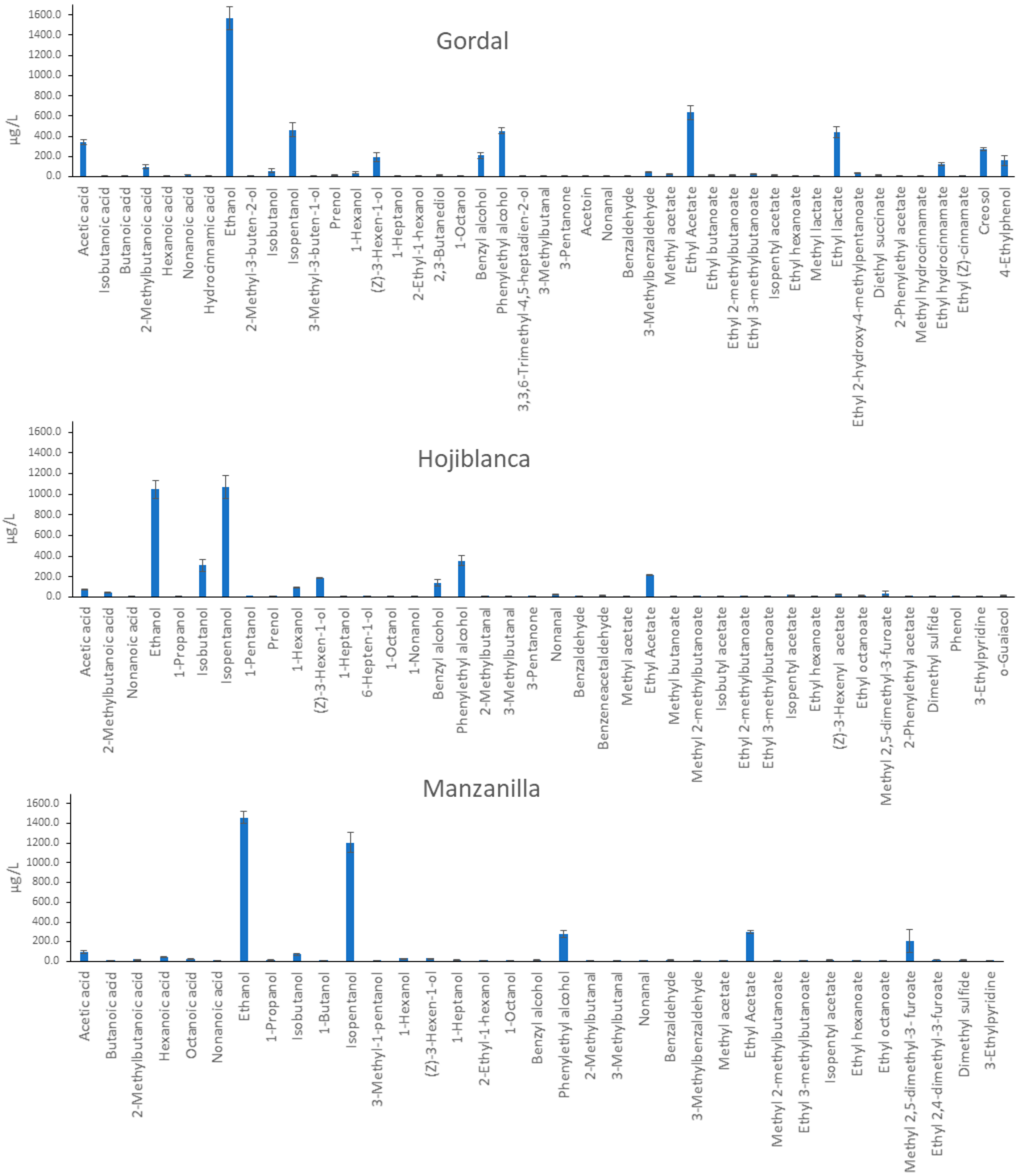

Figure S1. Volatile profiles in the fermenting brines of olives from the Gordal, Hojiblanca and Manzanilla cultivars processed in the natural style at the end of fermentation (180 days). Values are means of 2 biological replicates. Error bars represent standard deviations (*n* = 4).
